# Research on equivalent thermal network modeling for rare-earth giant magnetostrictive transducer

**DOI:** 10.1038/s41598-022-22959-7

**Published:** 2022-10-27

**Authors:** Zhihe Zhang, Xin Yang, Yukai Chen

**Affiliations:** National Electric Power Conversion and Control Engineering Technology Research Center (Hunan University), Changsha, Hunan China

**Keywords:** Electrical and electronic engineering, Thermodynamics, Ferromagnetism, Actuators

## Abstract

Of crucial importance for giant magnetostrictive transducers (GMTs) design is to quickly and accurately analysis the temperature distribution. With the advantages of low calculation cost and high accuracy, thermal network modelling has been developed for thermal analysis of GMT. However, the existing thermal models have their limits to describe these complicated thermal behaviors in the GMTs: most of researches focused on steady-state which is incapable of capturing temperature variances;  the temperature distribution of giant magnetostrictive (GMM) rods is generally assumed to be uniform whereas the temperature gradient on the GMM rod is remarkable due to its poor thermal conductivity;  the non-uniform distribution of GMM’s losses is seldom introduced into thermal model. Therefore, a transient equivalent thermal network (TETN) model of GMT is established in this paper, considering the aforementioned three aspects. Firstly, based on the structure and working principle of a longitudinal vibration GMT, thermal analysis was carried out. Following this, according to the heat transfer process of GMT, the TETN model was established and the corresponding model parameters were calculated. Finally, the accuracy of the TETN model for the temporal and spatial analysis of the transducer temperature are verified by simulation and experiment.

## Introduction

Giant magnetostrictive material (GMM), namely Terfenol-D, has the merits of large magnetostriction and high energy density. These unique characteristics can be exploited to enable the development of the giant magnetostrictive transducer (GMT) which can be used in a broad range of applications, such as under-water acoustic transducer, micro-motors, linear actuators and so on^1,2^.

Of particular concern is the possible overheating of underwater GMTs, which generate considerable heat due to their high dissipated power density when they are driven at full power and long excitation time^3,4^. In addition, the output characteristics of the GMT are closely related to temperature because of the large thermal expansion coefficient and its high sensitivity to external temperature^5–8^. Looking through the technical publications, the methods to face up the GMT thermal analysis can be divided into two main categories^9^: numerical and lumped parameter methods. The finite element method (FEM) is one of the most commonly used numerical analysis methods. Xie et al.^10^ used FEM to model heat source distribution of the giant magnetostrictive actuator and realized the temperature control of the actuator and the design of the cooling system. Zhao et al.^11^ created a coupled turbulent flow field and temperature field FEM simulation and constructed a GMM intelligent component temperature control device based on the FEM simulation results. However, FEM is very demanding in terms of model setup and computational time. For this reason, FEM is considered a valuable support for offline computations, typically during the transducer-design stage.

A lumped parameter method, often referred to as a thermal network model, is widely used in thermal dynamic analysis by virtue of its simple mathematical form and fast calculation speed^12–14^. This method has played an essential role in solving the thermal limitation problem of motors^15–17^. Mellor^18^ used an improved T-equivalent thermal circuit for the first time to simulate the heat transfer process of a motor. Verez et al.^19^ established a three-dimensional thermal network model for axial flux permanent magnet synchronous machines. Boglietti et al.^20^ proposed four thermal network models of different complexities for prediction of stator-winding short-term thermal transient. Finally, Wang et al.^21^ established the detailed equivalent thermal circuits for each component of the permanent magnet synchronous machine, and summarized the thermal resistance equations. The error can be controlled within 5% under rated conditions.

In the 1990s, the thermal network model began to be applied to low-frequency and high-power transducers. Dubus et al.^22^ built a thermal network model to describe heat transfer in double-ended longitudinal vibrator and class IV flextensional transducer at the steady state. Anjanappa et al.^23^ used the thermal network model to conduct a two-dimensional steady-state thermal analysis of a magnetostrictive mini-actuator. Zhu et al.^24^ established the steady-state equivalent thermal resistance and displacement calculation model of a GMT in order to study the relationship between the thermal deformation of Terfenol-D and the parameters of GMT.

Compared with that in the motor application, the GMT temperature estimation is more complicated. Most motor components considered at the same temperature have been usually simplified to a single node due to the excellent thermal and magnetic conductivity of the materials used^13,19^. Nevertheless, because of the poor thermal conductivity of GMM, the assumption of uniform temperature distribution is no longer valid. In addition, GMM has a very low permeability so that the heat generation resulted from magnetic losses is normally non-uniform along the GMM rod. Further-more, most of researches focused on steady-state modelling which is incapable of capturing temperature variances during the operation of GMTs.

To cope with the aforementioned three technical challenges, this paper takes the longitudinal vibration GMT as the research object, and accurately models different components of the transducer, especially the GMM rod. The complete transient equivalent thermal network (TETN) model of the GMT is established. A FEM model and an experimental platform were built to verify the accuracy and effectiveness of the TETN model for the temporal and spatial analysis of the temperature of the transducer.

## Longitudinal vibration GMT

### Structure and operating principle of the longitudinal vibration GMT

**Figure 1:**
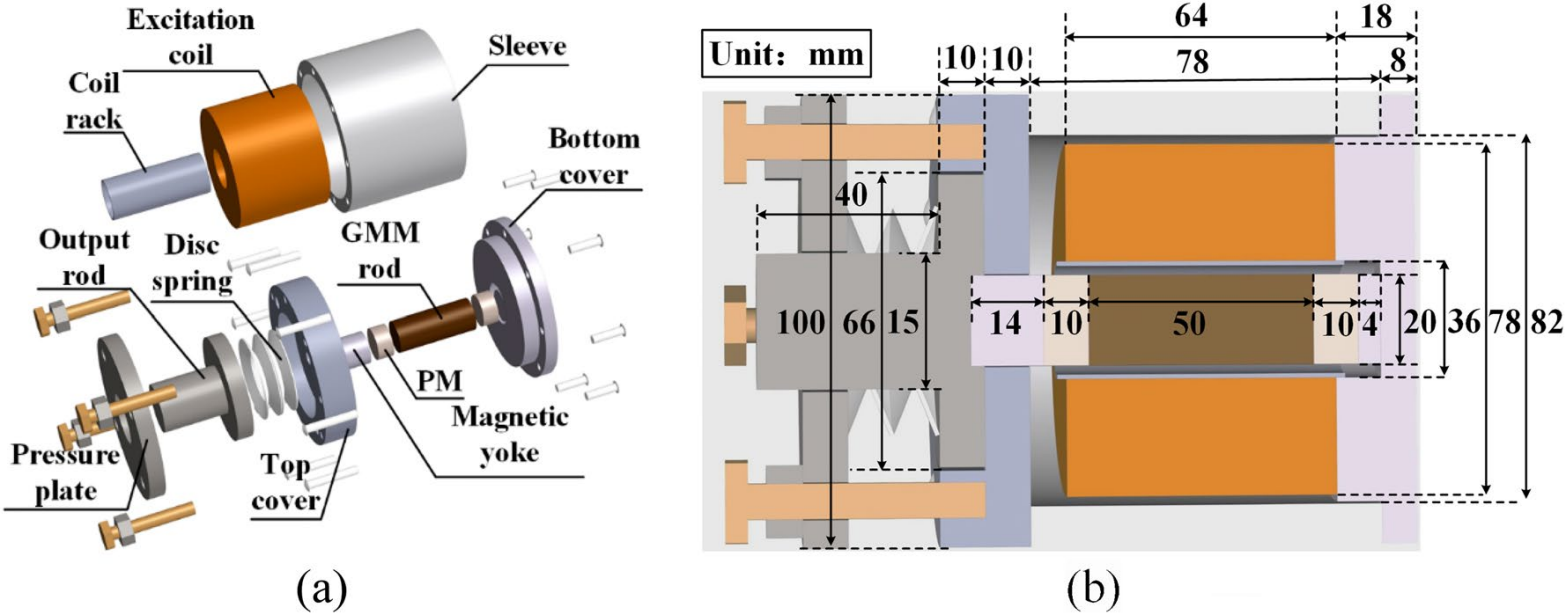
Longitudinal vibration GMT: (**a**) configuration diagram, (**b**) dimension diagram.

The structure and geometry dimensions of longitudinal vibration GMT are shown in Fig. 1a and b, respectively.

The main components include the GMM rod, the excitation coil, the permanent magnets (PM), the magnetic yokes, the cover plates, the sleeve, and the disc spring. The excitation coil and PMs provide alternating and dc-biased magnetic fields for the GMM rod respectively. The magnetic yokes and shell composed of cover plates and sleeve are all made of soft iron, DT4, with a high magnetic conductivity. Together with the GMM rod and PMs, a closed magnetic circuit is formed. The output rod and the pressure plate are made of non-magnetic 304 stainless steel. With the disc spring, a stable prestress to the rod can be applied. When an alternating current is passed through the excitation coil, the GMM rod would vibrate accordingly.

### Thermal analysis of the longitudinal vibration GMT

**Figure 2:**
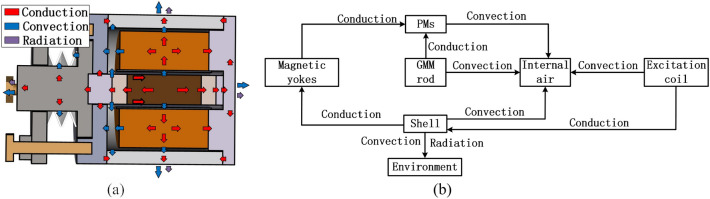
Heat transfer process in the longitudinal vibration GMT: (**a**) Heat flow diagram, (**b**) Main paths for heat transfer.

In Fig. 2, the heat transfer process inside the GMT is illustrated. The GMM rod and excitation coil are the two main heat sources of the GMT. The coil transfers its heat to the shell by convection through the internal air and towards the cover plate by conduction. The GMM rod would generate magnetic losses under the action of the alternating magnetic field, and transfers the heat to the shell by convection through the internal air and to the PMs and magnetic yokes by conduction. The heat transferred to the shell is then dissipated outwards the environment by convection and radiation. When the heat generated is equal to the heat transferred, the temperature of each component of the GMT reaches at a steady state.

Except for the heat generated on the excitation coil and the GMM rod, all components within the closed magnetic circuit will also suffer from magnetic losses. Therefore, the PMs, magnetic yokes, cover plates and sleeve are all laminated to suppress the magnetic losses for the GMT.

## Transient equivalent thermal network modelling for the longitudinal vibration GMT

The main steps of building a TETN model for thermal analysis of the GMT are as follows^25^: firstly, lump together components that have similar temperatures and represent each as a single node in the network; then, these nodes are coupled with appropriate heat transfer expressions, representing conduction and convection heat transfer between nodes. Meanwhile, the corresponding heat source and heat capacity of each component are connected in parallel between the node and zero voltage of common ground to build the equivalent heat network model. The next step is to calculate the thermal network parameters of each component in the model, including thermal resistances, heat capacities, and power losses. Finally, the TETN model is implemented in SPICE for simulation. And the temperature distribution in each component of the GMT and its variation in the time domain can be obtained.

### Basic assumptions of transient equivalent thermal network modelling

For the convenience of modelling and calculation, it is necessary to simplify the thermal model and ignore the boundary conditions that have little impact on the results^18,26^. The TETN model proposed in this paper is established on the basis of the following assumptions:

1. Only consider the losses of rod and coil, and ignore the loss of other components.
2. The influence of temperature on the thermal parameters of different materials is ignored^24,27^.
3. Radiation heat transfer is ignored^28^. Only conduction and convective heat transfer are considered.
4. The contact thermal resistance between different components is ignored.

### Establishment of transient equivalent thermal network model

In GMTs that have randomly-wound windings, it is impossible or undesirable to model the position of each individual conductor. In the past, various modeling strategies have been developed to model the heat transfer and temperature distribution within a winding: (1) composite thermal conductivity; (2) direct equations bases on conductor geometries; (3) T-equivalent thermal circuit^29^.

The composite thermal conductivity and direct equations can be considered more accurate solutions than T-equivalent circuit but they depend on several factors, such as the materials, conductor geometries, and residual air quantity in windings, which are difficult to determine^29^. On the contrary, the T-equivalent thermal circuit, although as an approximate model, is more convenient^30^. It can be applied to the excitation coil in the longitudinal vibration GMT.

**Figure 3:**
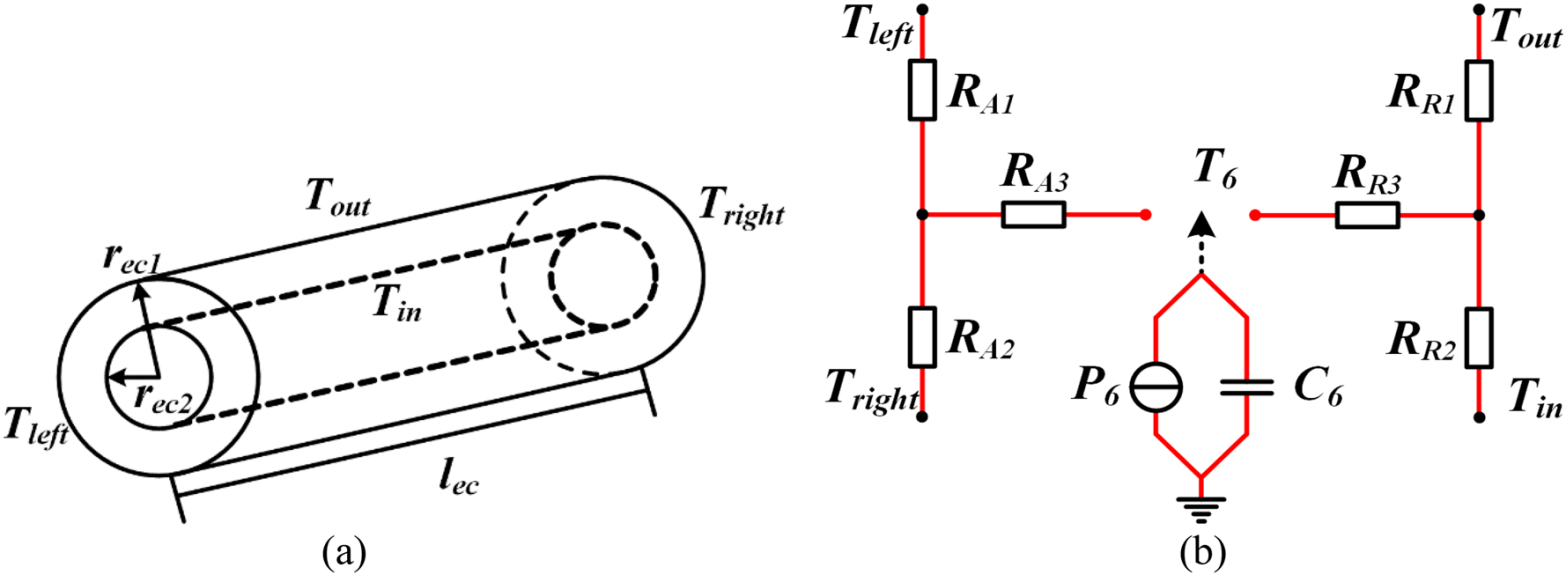
Excitation coil and its T-equivalent thermal circuit: (**a**) General hollow cylindrical component, (**b**) Independent axial and radial T-equivalent thermal circuits.

A general hollow cylindrical component used to represent the excitation coil and its T-equivalent thermal circuit that is derived from the solutions of the heat conduction equations are shown in Fig. 3. It is assumed that the heat flows inside excitation coil in the radial and axial directions are independent. The circumferential heat flow is ignored. In each T-equivalent circuit, two of the terminals represent the appropriate surface temperatures of the component, and the third terminal *T*_*6*_ represents the average temperature of the component. The losses *P*_*6*_ of the component are injected as a point source into the mean temperature node which are calculated in “Heat loss calculation of the excitation coil”. In the case of transient modeling, the heat capacity *C*_*6*_, which is given by Eq. (1), is also added to the mean temperature node.

1 $$C_{6} = c_{ec} \rho_{ec} V_{ec}$$

where *c*_*ec*_, *ρ*_*ec*_ and *V*_*ec*_ represents the specific heat capacity, the density and the volume of the excitation coil.

The thermal resistances for T-equivalent thermal circuit of the excitation coil with the length *l*_*ec*_, thermal conductivity *λ*_*ec*_, outer radius *r*_*ec1*_, and inner radius *r*_*ec2*_ are presented in Table 1.

**Table 1:** Thermal resistances for T-equivalent thermal circuit of the excitation coil^18^.

R	Radial thermal resistance	R	Axial thermal resistance
R_R1_	$$\frac{1}{{4\pi \lambda_{ec} l_{ec} }}\left( {1 - \frac{{2r_{ec2}^{2} \ln \left( {{\raise0.7ex\hbox{${r_{ec1} }$} \!\mathord{\left/ {\vphantom {{r_{ec1} } {r_{ec2} }}}\right.\kern-\nulldelimiterspace} \!\lower0.7ex\hbox{${r_{ec2} }$}}} \right)}}{{\left( {r_{ec1}^{2} - r_{ec2}^{2} } \right)}}} \right)$$	R_A1_	$$\frac{{l_{ec} }}{{2\pi \lambda_{ec} \left( {r_{ec1}^{2} - r_{ec2}^{2} } \right)}}$$
R_R2_	$$\frac{1}{{4\pi \lambda_{ec} l_{ec} }}\left( {\frac{{2r_{ec1}^{2} \ln \left( {{\raise0.7ex\hbox{${r_{ec1} }$} \!\mathord{\left/ {\vphantom {{r_{ec1} } {r_{ec2} }}}\right.\kern-\nulldelimiterspace} \!\lower0.7ex\hbox{${r_{ec2} }$}}} \right)}}{{\left( {r_{ec1}^{2} - r_{ec2}^{2} } \right)}} - 1} \right)$$	R_A2_	$$\frac{{l_{ec} }}{{2\pi \lambda_{ec} \left( {r_{ec1}^{2} - r_{ec2}^{2} } \right)}}$$
R_R3_	$$\frac{{{ - }\left( {r_{ec1}^{2} + r_{ec2}^{2} } \right)}}{{8\pi \lambda_{ec} l_{ec} \left( {r_{ec1}^{2} - r_{ec2}^{2} } \right)}}\left( {\frac{{2r_{ec1}^{2} r_{ec2}^{2} \ln \left( {{\raise0.7ex\hbox{${r_{ec1} }$} \!\mathord{\left/ {\vphantom {{r_{ec1} } {r_{ec2} }}}\right.\kern-\nulldelimiterspace} \!\lower0.7ex\hbox{${r_{ec2} }$}}} \right)}}{{\left( {r_{ec1}^{2} - r_{ec2}^{2} } \right)}}} \right)$$	R_A3_	$$\frac{{ - l_{ec} }}{{6\pi \lambda_{ec} \left( {r_{ec1}^{2} - r_{ec2}^{2} } \right)}}$$

### Equivalent thermal circuit of GMM rod

T-equivalent circuit has also been proved accurate for other cylindrical heat sources^13^. As the main heat source of GMT, the temperature distribution of GMM rod is non-uniform due to its low thermal conductivity and the discrepancy is particularly pronounced along the axial of the rod. On the contrary, the radial non-uniformity can be ignored because the radial heat flow of GMM rod is far less than the radial heat flow^31^.

**Figure 4:**
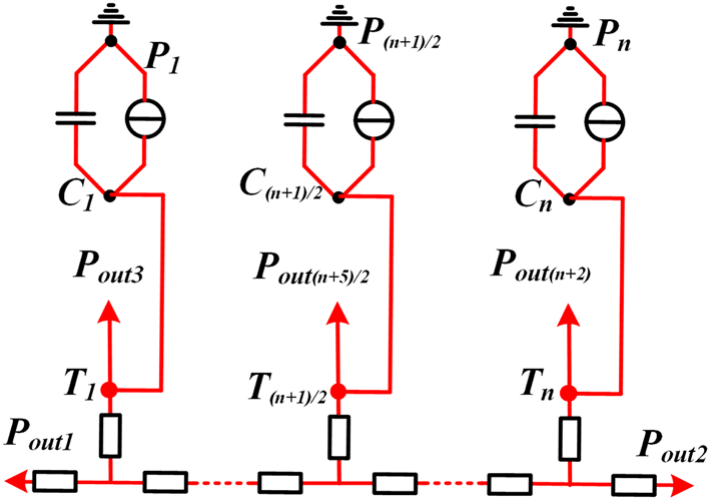
Thermal network model with n T-equivalent network circuit of GMM rod.

In order to accurately represent the axial discretization level of the rod and obtain the highest temperature, the GMM rod is represented by *n* nodes equally spaced in the axial direction, and the number of nodes *n* for GMM rod modeling must be equal to an odd number. The thermal circuit with *n* T-equivalent axial thermal circuits is presented in Fig. 4.

**Figure 5:**
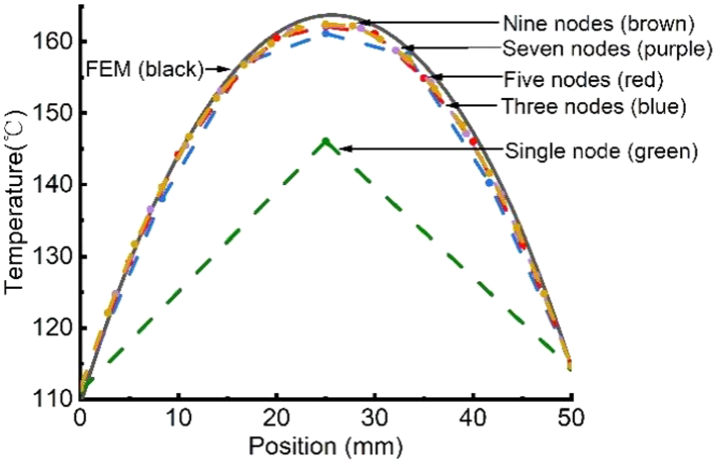
Temperature distribution of the GMM rod in FEM and thermal network model with different complexity.

In order to determine the number of nodes *n* for GMM rod modeling, the FEM result is illustrated in Fig. 5 as a benchmark. The number of nodes *n* is tuned in the thermal circuit of GMM rod as shown in Fig. 4. Each node can be model as a T-equivalent circuit. Compared the result of FEM, it can be seen from Fig. 5 that one or three nodes cannot accurately reflect the temperature distribution of the GMM rod (a length of around 50 mm) in the GMT. When* n* is increased to five, the simulation results would be much improved and close to FEM. Further increase of *n* can also produce even better results but it will occur at the expense of increased computation time. Therefore, five nodes are selected in this paper to model the GMM rod.

**Figure 6:**
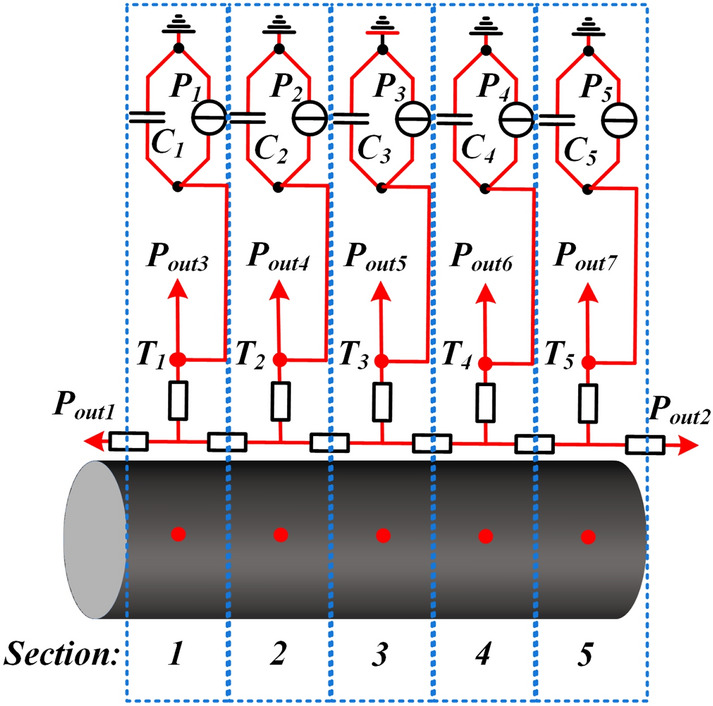
Equivalent thermal circuit with 5 nodes for the GMM rod in the GMT.

Based on the above comparative analysis, the accurate thermal circuit of GMM rod is presented in Fig. 6. *T*_*1*_ ~ *T*_*5*_ represent the mean temperatures of the five sections (Sections 1 ~ 5) of the rod. *P*_*1*_ ~ *P*_*5*_ represent the total thermal powers in different regions of the rod respectively, which are discussed in detail in the next chapter. *C*_*1*_ ~ *C*_*5*_ represents the heat capacities of different regions, which can be calculated by

2 $$C_{1} = C_{2} = C_{3} = C_{4} = C_{5} = {{\left( {c_{rod} \rho_{rod} V_{rod} } \right)} \mathord{\left/ {\vphantom {{\left( {c_{rod} \rho_{rod} V_{rod} } \right)} 5}} \right. \kern-\nulldelimiterspace} 5}$$

where *c*_*rod*_, *ρ*_*rod*_ and *V*_*rod*_ represents the specific heat capacity, the density and the volume of the GMM rod.

Applying the same method as for the excitation coil, the transfer thermal resistances of the GMM rod in Fig. 6 can be calculated by

3 $$R_{A5} = R_{A7} = R_{A9} = R_{A11} = 2R_{A4} = 2R_{A13} = \frac{{l_{rod} }}{{5\pi \lambda_{rod} r_{rod}^{2} }}$$

4 $$R_{A6} = R_{A8} = R_{A10} = R_{A12} = R_{A14} = \frac{{ - l_{rod} }}{{30\pi \lambda_{rod} r_{rod}^{2} }}$$

where *l*_*rod*_, *r*_*rod*_ and *λ*_*rod*_ represents the length, the radius and the thermal conductivity of the GMM rod.

### Equivalent thermal circuit of other regions

For the longitudinal vibration GMT studied in this paper, the rest components and internal air can be modelled with single-node configurations.

These regions can be considered to consist of one or more cylinders. Pure conduction heat transfer couplings in the cylindrical component are determined from Fourier’s Law of heat conduction as

5 $${\text{Radial}}\;{\text{conduction}}: R_{R,nhs} = \frac{{\ln \left( {{\raise0.7ex\hbox{${r_{nhs1} }$} \!\mathord{\left/ {\vphantom {{r_{nhs1} } {r_{nhs2} }}}\right.\kern-\nulldelimiterspace} \!\lower0.7ex\hbox{${r_{nhs2} }$}}} \right)}}{{2\pi \lambda_{nhs} l_{nhs} }}$$

6 $${\text{Axial}}\;{\text{conduction}}: R_{A,nhs} = \frac{{l_{nhs} }}{{\pi \lambda_{nhs} \left( {r_{nhs1}^{2} - r_{nhs2}^{2} } \right)}}$$

where λ_*nhs*_ is the thermal conductivity of the material, *l*_*nhs*_ is an axial length, and *r*_*nhs1*_ and *r*_*nhs2*_ are the outer and inner radius of the heat transfer component, respectively.

**Figure 7:**
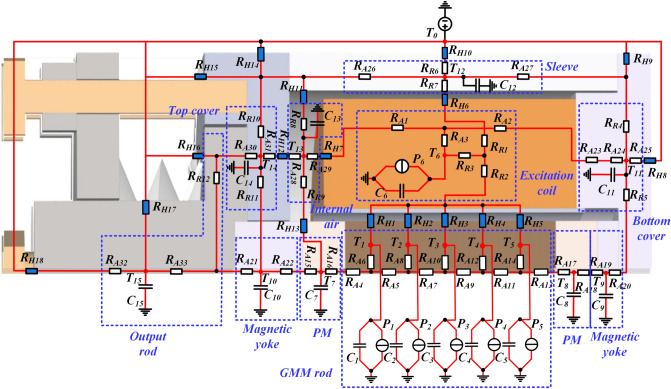
Equivalent thermal network model of the longitudinal vibration GMT.

Equation (5) is used for calculation of the radial thermal resistances of these regions represented by *R*_*R4*_ to *R*_*R12*_ in Fig. 7. Meanwhile, Eq. (6) is used for calculation of the axial thermal resistances represented by *R*_*A15*_ to *R*_*A33*_ in Fig. 7.

The heat capacity for the single-node thermal circuit of the aforementioned regions, including *C*_*7*_ to *C*_*15*_ in Fig. 7 can be given by

7 $$C = c_{nhs} \rho_{nhs} V_{nhs}$$

where *ρ*_*nhs*_, *c*_*nhs*_ and *V*_*nhs*_ represent the length, specific heat capacity density and volume, respectively.

### Convection thermal resistance calculation

Convective heat transfers across the internal air within the GMT as well as between the shell surface and the ambient is modelled with a single thermal conductive resistance as follows:

8 $$R_{con} = \frac{1}{hA}$$

where *A* represents the contact surface and *h* is the heat transfer coefficient. Some typical *h* used in the thermal systems are listed in Table 2^32^. According to Table 2, heat transfer coefficients of thermal resistances *R*_*H8*_*–R*_*H10*_ and *R*_*H14*_*–R*_*H18*_ in Fig. 7, which represented convection between the GMT and the ambient, take the constant value of 25 W/(m^2^ K). The rest heat transfer coefficients is set to 10 W/(m^2^ K).

**Table 2:** Heat transfer coefficient for some common fluids [W/(m^2^ K)]^32^.

Free convection-air	5–25
Free convection-water	20–100
Forced convection-air	10–200
Forced convection-water	50–10,000
Boiling water	3000–100,000
Condensing water vapor	5000–10,000

### Complete realization of the equivalent thermal network model of the longitudinal vibration GMT

According to the internal heat transfer process shown in Fig. 2, the complete TETN model of the transducer given in Fig. 7.

As can be seen in Fig. 7, the longitudinal vibration GMT was divided into 16 nodes, which were shown in red dots. The temperature nodes depicted in this model correspond to the average temperatures of their respective components. The ambient temperature is *T*_*0*_, the GMM rod temperature *T*_*1*_ ~ *T*_*5*_, the excitation coil temperature *T*_*6*_, the PMs temperature *T*_*7*_ and *T*_*8*_, the magnetic yokes *T*_*9*_ ~ *T*_*10*_, the shell temperature *T*_*11*_ ~ *T*_*12*_ and *T*_*14*_, the internal air temperature *T*_*13*_, and the output rod temperature is *T*_*15*_. Moreover, each node is connected to the thermal ground potential via *C*_*1*_ ~ *C*_*15*_ which respectively represent the thermal capacity of each region. *P*_*1*_ ~ *P*_*6*_ represent the total thermal powers of the GMM rod and excitation coil respectively. In addition, 54 thermal resistances are used to represent the conduction and convection heat transfer resistances between adjoining nodes, which have been calculated in the previous sections. Table 3 shows the different thermal properties of the materials which compose the transducer.

**Table 3:** Thermophysical properties of the main materials in longitudinal vibration GMT^33–35^.

Component	Thermal conductivity [W/(m K)]	Specific heat [J/(kg K)]	Density (kg/m^3^)	Note
GMM rod	13.5	350	9250	Terfenol-D
Excitation coil	401	486	8933	Copper
PM	8.9	514.86	7400	SmCo
Shell/permeability block	54.7	574	7870	DT4
Output rod	17.2	500	8000	SUS304

## Heat losses calculation of the longitudinal vibration GMT

Accurate evaluation of the amount of losses and their distribution is of major importance in performing a reliable thermal modeling. The heat losses generated in the GMT can be divided into magnetic loss of GMM rod, joule loss of excitation coil, mechanical loss and additional loss. The additional loss and mechanical loss accounted for are relatively small and can be ignored^36^.

### Heat loss calculation of the excitation coil

The AC resistance of the excitation coil includes: DC resistance *R*_*dc*_ and skin resistance *R*_*s*_. For a sinusoidal alternating current with a Root Mean Square (RMS) value of *I*, the total thermal power *P*_*6*_ of the coil is given by^35^

9 $$P_{6} = I^{2} R_{ac} = I^{2} \sqrt {R_{dc}^{2} + R_{s}^{2} }$$

The DC resistance *R*_*dc*_ can be calculated by

10 $$R_{dc} = \frac{{\rho_{Cu} l_{Cu} }}{{\pi r_{Cu}^{2} }} = \frac{{N\rho_{Cu} \left( {r_{ec1} + r_{ec2} } \right)}}{{r_{Cu}^{2} }}$$

The resistance *R*_*s*_ due to the skin effect is given by

11 $$R_{s} = \frac{{l_{Cu} }}{{2\pi r_{Cu} }}\sqrt {\pi f\mu_{Cu} \rho_{{_{Cu} }} } = \frac{{N\left( {r_{ec1} + r_{ec2} } \right)}}{{2r_{Cu} }}\sqrt {\pi f\mu_{Cu} \rho_{{_{Cu} }} }$$

where *f* and *N* are the frequency of the excitation current and the number of turns. *l*_*Cu*_ and *r*_*Cu*_ represent the inner and outer radius of the coil, the length of the coil and the radius of the copper magnetic wire which is defined by its AWG (American Wire Gauge) number. *ρ*_*Cu*_ is resistivity of its core. *μ*_*Cu*_ is permeability of its core.

### Heat loss calculation of the GMM rod

**Figure 8:**
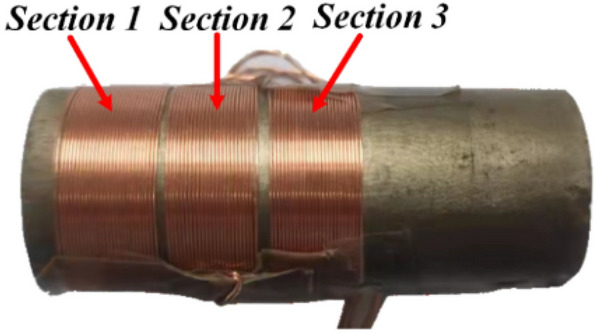
Positions of induction coils on the GMM rod.

The actual magnetic field within the excitation coil, a solenoid, is non-uniform across the length direction of the rod. The discrepancy is particularly pronounced due to the low magnetic conductivity of GMM rod and PM. But it is longitudinally symmetric. The magnetic field distribution directly determines the distribution of magnetic loss of the GMM rod. Therefore, to reflect the actual loss distribution, three sections of the rod shown in Fig. 8 are taken for measurement.

**Figure 9:**
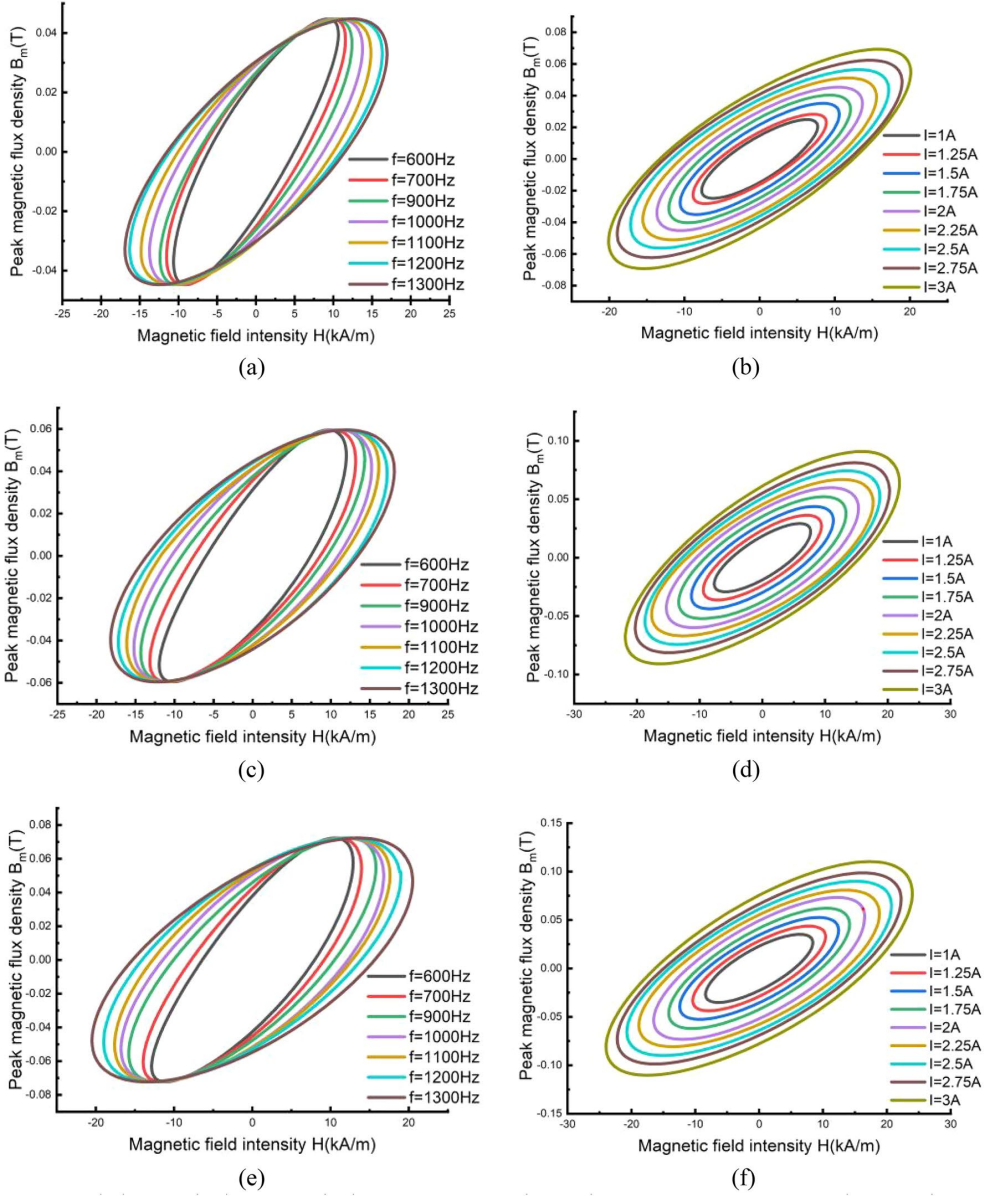
Measured dynamic hysteresis loops: (**a**) Section 1/5: B_m_ = 0.044735 T, (**b**) Section 1/5: f_m_ = 1000 Hz; (**c**) Section 2/4: B_m_ = 0.05955 T, (**d**) Section 2/4: f_m_ = 1000 Hz, (**e**) Section 3: B_m_ = 0.07228 T, (f) Section 3: f_m_ = 1000 Hz.

Magnetic loss can be obtained by measuring the dynamic hysteresis loop^37^. The dynamic hysteresis loops of three sections were measured based on the experimental platform shown in Fig. 11. Under the condition of the GMM rod temperature stable below 50 °C, a programmable ac power supply (Chroma 61512) drives the excitation coil over a range of frequencies with a test current to produce a magnetic field and the resulting flux density is derived by integrating the voltage induced in the induction coils attached to the GMM rod as shown in Fig. 8. Raw data was downloaded from the memory hicorder (Daily MR8875-30) and processed in MATLAB software to derive the measured dynamic hysteresis loops shown in Fig. 9.

According to literature^37^, the total magnetic loss per unit volume *P*_*v*_ of GMM rod can be calculated from:

12 $$P_{v} = A_{BH} \cdot f_{m} ({W \mathord{\left/ {\vphantom {W {m^{3} }}} \right. \kern-\nulldelimiterspace} {m^{3} }})$$

where *A*_*BH*_ is the measured area within the B-H curve at the magnetic field frequency *f*_*m*_ which is equal to the excitation current frequency *f*.

Based on the Bertotti loss separation method^38^, magnetic loss per unit mass of GMM rod *P*_*m*_ can be expressed as the sum of hysteresis loss *P*_*h*_, eddy current loss *P*_*e*_ and abnormal loss *P*_*a*_ (13):

13 $$P_{m} = P_{h} + P_{e} + P_{a} = k_{h} f_{m} B_{m}^{\alpha } + k_{e} f_{m}^{2} B_{m}^{2} + k_{a} f_{m}^{1.5} B_{m}^{1.5}$$

From the perspective of engineering analysis^38^, abnormal loss and eddy current loss can be combined into one term called total eddy current loss. Therefore, the loss calculation formula can be simplified to:

14 $$P_{m} = P_{h} + P_{c} = k_{h} f_{m} B_{m}^{2} + k_{c} f_{m}^{2} B_{m}^{2}$$

In Eqs. (13)–(14), *B*_*m*_ is the magnetic density amplitude of the excitation magnetic field. *k*_*h*_ and *k*_*c*_ are the hysteresis loss coefficient and the total eddy current loss coefficient.

According to literature^39^, based on simplified loss separation formula and loss test data, polynomial curve fitting method is adopted to mathematically obtain the hysteresis loss coefficient and total eddy current loss coefficient, which are listed as (12) and (13):

15 $$k_{h} \left( {B_{m} } \right) = a_{0} + a_{1} B_{m} + a_{2} B_{m}^{2}$$

16 $$k_{c} \left( f \right) = b_{0} + b_{1} f_{m} + b_{2} f_{m}^{2} + b_{3} f_{m}^{3}$$

where *a*_*0*_, *a*_*1*_, *a*_*2*_, *b*_*0*_, *b*_*1*_, *b*_*2*_ and *b*_*3*_ are the relevant parameters of material loss. According to the fitted experimental data, the seven loss-related parameters in different sections were obtained for different sections (Fig. 6) as shown in Table 4.

**Table 4:** Parameter values of the magnetic loss calculation model.

	Section 1/5	Section 2/4	Section 3
*a*_*0*_	− 548.3625706	− 244.8508898	66.33900392
*a*_*1*_	− 80.56	− 111.9	− 13.64
*a*_*2*_	− 40.78	− 130.3	− 223.1
*b*_*0*_	2.742326872	1.279960747	− 0.217058485
*b*_*1*_	− 0.004678645	− 0.002101967	0.000431054
*b*_*2*_	3.51386E-06	1.5518E−06	− 3.05681E−07
*b*_*3*_	− 9.58915E−10	− 4.16784E−10	8.16362E−11

Table 5 shows the magnetic loss values *P*_*1*_ ~ *P*_*5*_ in different sections of the GMM rod by the fitted loss separation formula at different current levels.

**Table 5:** Values of the magnetic power loss at different positions of the GMM rod.

Frequency (Hz)	Current (A)	Magnetic loss (W)			
		*P*_*1*_/*P*_*5*_	*P*_*2*_/*P*_*4*_	*P*_*3*_	Total
1000	1.5	2.42	3.51	4.42	16.28
	2	3.96	6.35	8.43	29.05
	2.5	5.01	7.82	10.19	35.85
	3	9.00	13.65	18.40	63.7

It can be seen from Table 4 that the magnetic loss at the GMM rod ends are far less than that at the center, which proves the obvious non-uniformity of magnetic loss along the axial direction of the rod. Meanwhile, the non-uniformity is continuously amplified with the increase of the excitation current. The non-uniform loss distribution of GMM rod significantly affects the accuracy of whatever thermal analysis approach, especially for low frequency, high power GMT.

## Validation of the proposed equivalent thermal network model

### FEM verification

**Figure 10:**
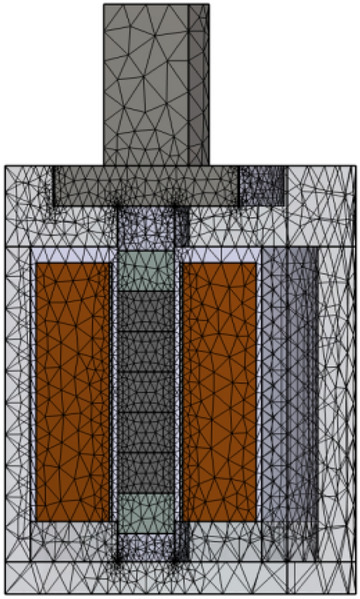
Finite element model of longitudinal vibration GMT.

To better verify the accuracy of the TETN model for the temperature distribution of the GMM rod, the longitudinal vibration GMT was modeled by employing COMSOL Multiphysics software shown in Fig. 10. A half symmetry sector with symmetry boundary conditions and a volume free meshing generated by only pyramid (tetrahedral) elements type was used. Complete mesh consists of 108,924 domain elements, 23,976 boundary elements, and 2448 edge elements completely.

**Figure 11:**
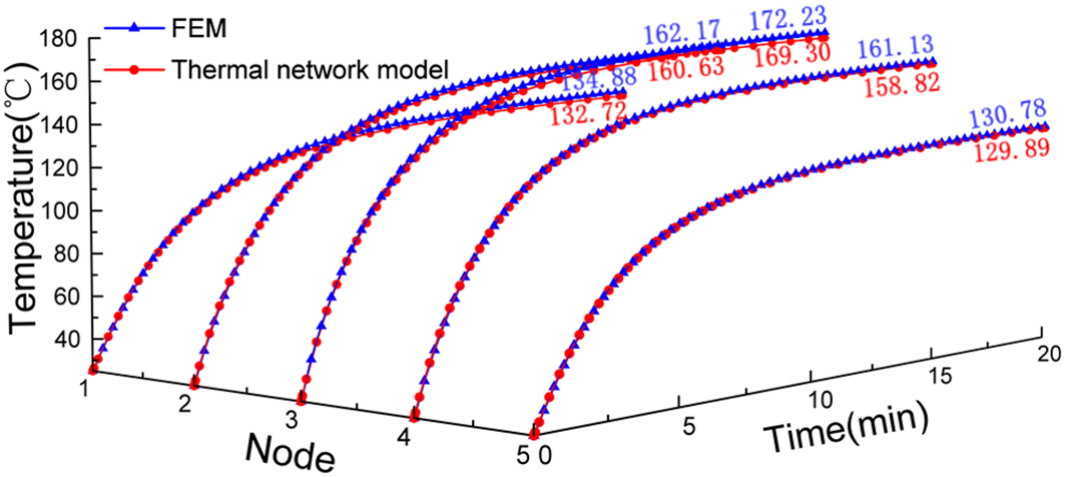
Comparison of the GMM rod temperature at different positions obtained from the FEM simulation and the TETN model.

The GMM rod temperature *T*_*1*_ ~ *T*_*5*_ obtained from the FEM simulation and the TETN model are all compared in Fig. 11 under *I* = 3A and *f* = 1000 Hz.

As Fig. 11 shows, the temperature is lowest at node 5. The TETN model produces a smaller temperature than FEM with an error of around 0.89 °C at the steady state. With the increased of node temperature, the differences between the TETN model and FEM increased to 2.31 °C and 2.93 °C respectively at node 4 and node 3. Figure 11 shows a good agreement between the simulation results of the TETN model and FEM with a small error of below 2%, which indicates that the proposed model achieves an accurate simulation result for GMM rod temperature distribution.

### Experimental verification

**Figure 12:**
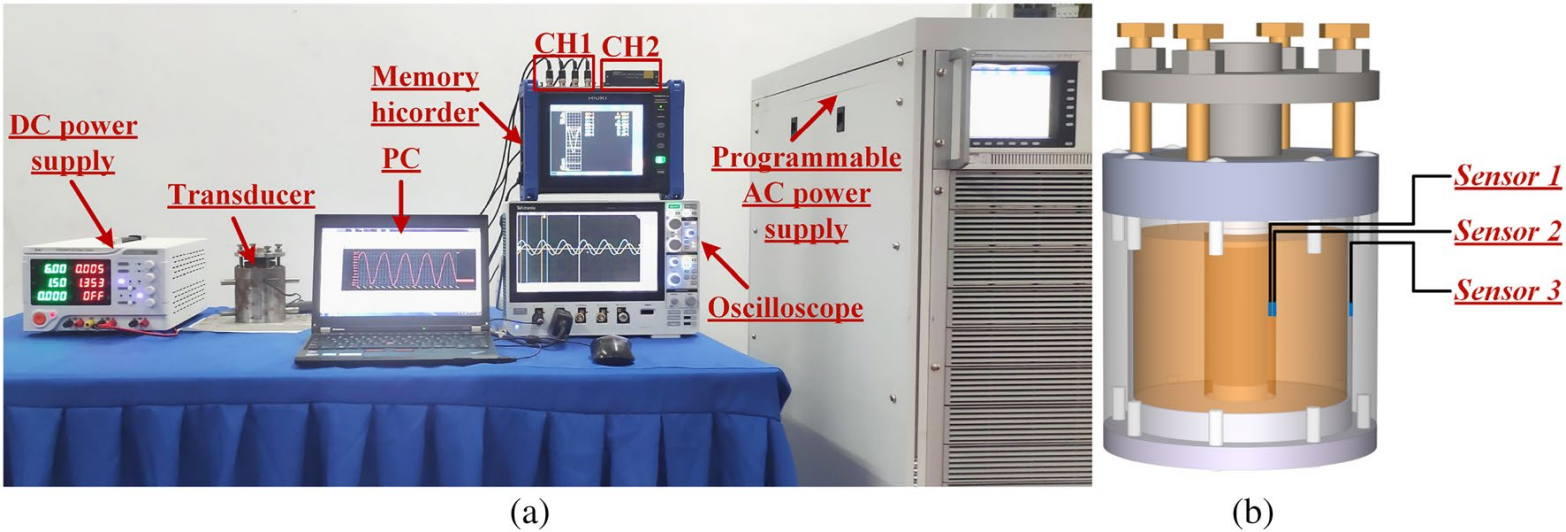
Temperature rise experimental platform for longitudinal vibration GMT: (**a**) Experimental platform, (**b**) Selected test positions.

IN order to validate the effectiveness and accuracy of the proposed model, a temperature rise experimental platform has been built as shown in Fig. 12a. A programmable ac power supply (Chroma61512) is chosen to supply and control excitation current of the longitudinal vibration GMT. K-type temperature sensors are used for real-time collection of the temperature at different positions. An oscilloscope (Tektronix MDO34) is used for monitoring voltage and current, while a memory hicorder (Daily mr8875-30) for data storage. The computer gets data from the memory hicorder, and is used to process data.

Starting with the longitudinal vibration GMT at the ambient temperature, the test is performed for a time length of 20 min to avoid the GMM rod temperature reaching half of the Curie point, which would affect the reliable operation of the GMT. For comparison reasons, the central surface of the rod, the inner wall of excitation coil and shell temperatures are recorded during the experimental investigations.

To demonstrate the performance of the TETN model for temperature estimation, the temperatures of the central surface of the rod, the inner wall of the excitation coil and shell were measured when *I* range from 1.5 to 3 A, as show in Fig. 12b.

Final results of the proposed model, FEM and experimental measurement at various positions are presented in Table 6. The maximum final temperature differences seen between the results of TETN simulations and the measured data is 3.46 °C in the central surface of GMM rod, 2.03 °C in the inner wall of excitation coil and 1.44 °C in the inner wall of shell respectively.

**Table 6:** Comparison of calculated and measured final temperatures at various locations.

Frequency (Hz)	Current (A)	The GMM rod			The excitation coil			The shell		
		TETN (℃)	FEM (℃)	Measured (℃)	TETN (℃)	FEM (℃)	Measured (℃)	TETN (℃)	FEM (℃)	Measured (℃)
1000	1.5	67.6	68	69	47.7	47.8	48.7	27.3	27.4	26.9
	2	101.7	102.7	101.8	64.6	65.2	64.9	29.1	29.2	30.0
	2.5	140.2	141.4	143.3	88.5	90.4	90.3	31.2	31.4	31.9
	3	171.4	172.4	174.7	109.3	113	111.3	34	34.4	35.4

**Figure 13:**
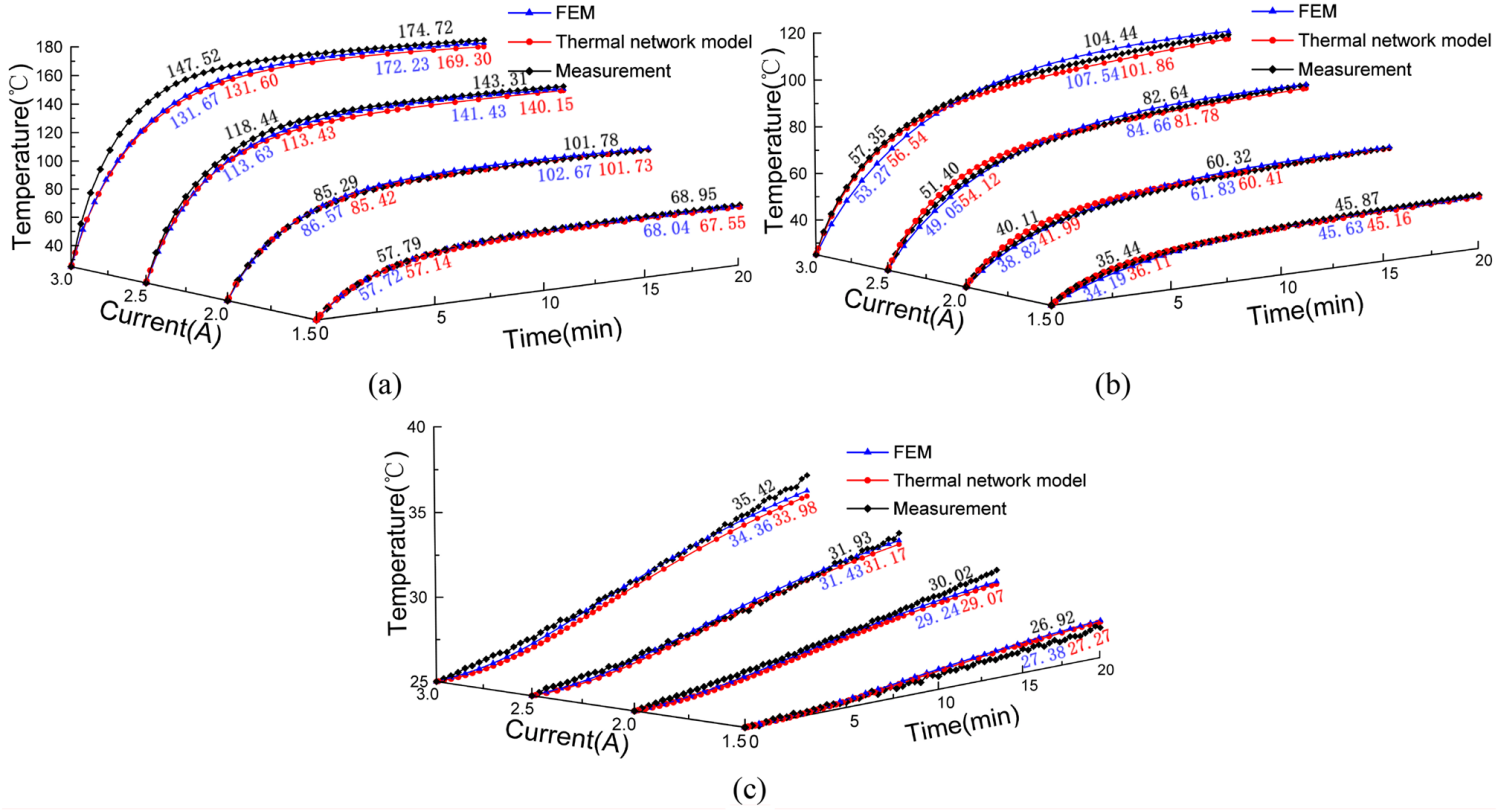
Comparison of measured values and model results of temperature rise at different positions of GMT: (**a**) The central surface of GMM rod, (**b**) The inner wall of excitation coil, (**c**) The inner wall of sleeve.

Figure 13 shows a time-based comparison between the measured and simulated results at three selected test positions. The simulated results for the GMM rod are comparable to the measured results with a slight deviation within 1.5 °C when the excitation current is 1.5 A and 2 A in Fig. 13a. With the increase of temperature, the differences between the measured and simulated results occurring during the initial transient period increase to 5.01 °C and 15.92 °C at *I* = 2.5 A and 3 A, which probably is attributed to the less accurate heat capacity value for GMM rod. Furthermore, the power losses are not constant at high temperatures in reality, which are assumed to be unchanged as measured at room temperature^39^.

As shown in Fig. 13b, the proposed model is more accurate in estimation of the excitation coil temperature. The maximum errors of the TETN model and experiment are within 3 °C. However, it is apparent that the temperature rise trend for the coil for FEM is quite different from the experiment. The main reason probably is that the excitation coil transfers its heat by convection through the internal air. Nevertheless, COMSOL cannot accurately describe the heat flux of internal air. In Fig. 13c, there is a good agreement between the measured and calculated results of the temperature of the inner wall of the shell. Whereas, with the increase of the excitation current, the measured temperature increases faster than the calculated temperature as the surface radiation of the shell which is assumed in the proposed model to be ignored becomes increasingly important in reality.

To sum up, the proposed thermal modeling method can be successfully applied to the low frequency, high power GMT. Optimization on the TETN model should be required to account for the radial heat transfer if the radial loss distribution of the GMM rod become very pronounced at higher operating frequency due to the increased eddy current effect.

## Conclusion

A TETN model has been established and applied to estimate the temperature distribution of a longitudinal vibration GMT in this paper. In particular, the distinctiveness of GMM rod being also as a heat source, and additionally the influence of temperature and heat generation distribution of the GMM rod are fully discussed and considered. In this paper, the complete procedure for the equivalent thermal circuits of each part of the GMT was reported and the corresponding model parameters were calculated in detail. The accuracy of this model in transducer temperature estimation was verified through FEM simulation and experiments. Furthermore, the error analysis at all selected test positions is discussed, which should shed light on the further improvement of the TETN model.

## Data Availability

The datasets generated during and/or analysed during the current study are available from the corresponding author on reasonable request.
